# Experiences with the Implementation of Cuban Health Cooperation Programs in Low and Middle-Income Countries: A Scoping Review

**DOI:** 10.12688/wellcomeopenres.23844.1

**Published:** 2025-03-28

**Authors:** Faith Njiriri, Moriasi Nyanchoka, Jacinta Nzinga, Benjamin Tsofa

**Affiliations:** 1 Health Economics Research Unit, KEMRI-Wellcome Trust Research Program, Nairobi Kenya, Nairobi, Kenya; 2 Department of Public Health, School of Human and Health Scienes, Pwani University, Kilifi, Kilifi County, Kenya; 3 Liverpool School of Tropical Medicine, Liverpool, England, UK; 4 Health Systems and Research Ethics Department, KEMRI-Wellcome Trust Research Program, Kilifi Kenya, Kilifi, Kenya

**Keywords:** Health cooperation, Human resources for health, Health systems, Primary health care, Universal health coverage, Low-and-middle income countries, Cuba

## Abstract

**Background:**

Health systems in low and middle-income countries (LMICs) face chronic Human Resources for Health (HRH) shortages. This is especially worse in rural and primary healthcare settings. The Cuban government since 1960s has been implementing a policy strategy for producing healthcare workers for export, to boost their economy. Several LMICs have since established health cooperation programs with Cuba to import health workers to address their shortages. This review aimed to examine the emergence, design, utility, outcomes, and lessons learned from the implementation of these programs.

**Methods:**

We conducted a scoping review using the Joanna Briggs Institute (JBI) methodology and searched for literature across four databases. Two independent reviewers screened the articles and selected relevant articles based on pre-defined criteria. We extracted data and synthesized findings using thematic analysis.

**Results:**

We included 71 articles after screening 3509 articles. Cuban health cooperation programs have been implemented in many LMICs in South America, Africa, Southeast Asia, and the Pacific region. These programs are formalized primarily through bilateral agreements and implemented as exchange initiatives. This involves importing Cuban healthcare workers and sending collaborating country students to study in Cuba. These programs aimed to address HRH shortages and maldistribution, inadequate training capacity, and respond to medical emergencies in the host countries. Cuban healthcare workers, primarily family physicians, within the host countries; are deployed in primary healthcare settings, increasing the rural health workforce, and improving healthcare access and outcomes. These programs have faced several challenges including opposition from local medical professionals, underutilization due to poorly coordinated recruitment, and language barrier.

**Conclusion:**

Cuban health cooperations in LMICs have shown diverse results based on their structures. Long-term comprehensive programs have proven to be more successful in boosting the healthcare workforce and enhancing health outcomes. Key factors for optimizing HRH health cooperation include effective collaborative decision-making and need-based deployment.

## Plain Language Summary

Health systems in many low and middle-income countries have inadequate numbers of healthcare workers, especially in rural areas and in primary healthcare settings. The Cuban government for many years has trained healthcare workers for export to boost its economy. Many countries have then collaborated with Cuba to send their healthcare workers to work in the collaborating LMICs to address these healthcare workers' shortages. This scoping review aimed to examine how these health cooperation programs between Cuba and LMICs have emerged, their intended goals, the implementation processes and lessons learned. The reviewers collected and analysed data from 71 articles. These health cooperation programs between Cuba and LMICs have been implemented in many countries in South America, Africa, Southeast Asia and the Pacific region. These collaborative programs aimed to address healthcare worker shortages and their uneven distribution between rural and urban areas, cater for inadequate training capacity and respond to medical emergencies such as Ebola and COVID-19 pandemic in the host countries. The programs have mainly been designed as exchange programs, importing Cuban healthcare workers and sending collaborating countries students to study in Cuba. The Cuban healthcare workers, within the host countries, have worked in rural areas, increasing the numbers of healthcare workers in these areas and contributing to improving healthcare access and outcomes. These programs faced several challenges such as opposition from the local healthcare workers, language barrier and underutilization due to poorly coordinated deployment. There is a need to involve key health stakeholders in decision-making and to ensure that healthcare workers are deployed where they are needed most.

## Introduction

Human resources for health (HRH) plays a crucial role in the effective functioning of health systems ^1^. The success of any country in achieving its health goals depends on adequate training, absorption into service, deployment, and retention of highly qualified healthcare workers ^2, 3^. Inadequate supply of skilled healthcare workers is a common problem facing most health systems globally. These HRH shortages are more severe in Low- and Middle-Income Countries (LMICs), especially in rural areas and primary healthcare settings ^4^. HRH shortages and skewed distribution of healthcare workers between urban and rural areas exacerbate health inequalities and pose a challenge to achieving universal health coverage (UHC). To address these health workforce shortages and maldistribution, different countries have adopted various interventions including the importation of healthcare workers.

Globally, the importation and exportation of healthcare workers are increasing, and many countries are increasingly relying on imported or in-migrant healthcare workers to fill critical HRH shortages ^5, 6^. As health workforce shortages continue to grow with a projected shortfall of 18 million by 2030, mostly in LMICs, health workforce mobility is likely to accelerate ^4^. The patterns of healthcare workers, importation, and exportation have often involved migration to the global north (South-North). However, over time the need for an adequate and adequately skilled health workforce has accelerated migration to the global south (South-South) ^7^.

Cuba has historically established one of the largest HRH export programs globally, with an estimated 325,000 Cuban healthcare workers having worked abroad as of 2020 ^8^. These programs have often involved Cuba exporting healthcare workers on multi-year contracts to countries with shortages to support the delivery of healthcare services in those collaborating countries. To enable this, Cuba invested heavily in health worker training, ensuring a steady supply and producing a surplus of healthcare workers for export. This has continuously enabled Cuba to export its surplus healthcare workers to other countries. Cuba, despite being a low-resource setting, has had an exemplary model health system globally. Cuba’s health system prioritizes preventive measures, community-focused care, and active citizens engagement, thus providing valuable insights for other LMICs aiming to enhance primary health care services and attain UHC ^9^.

Globally, Cuban health worker import programs have been implemented in different countries. The Cuba-Netherlands cooperation, for example, involved an exchange program between Dutch universities and Cuban health institutions. This involved Dutch healthcare professionals and students visiting Cuban healthcare facilities to learn from the Cuban healthcare system and its primary healthcare model. This was considered important by the Netherlands due to the high Non-Communicable Diseases (NCDs) morbidity ^10^. Cuba has also had long-term cooperation programs with countries such as Venezuela, where they exchanged Cuban healthcare workers to work in Venezuela in exchange for oil imports to Cuba from Venezuela ^11^. Cuban healthcare workers have also been deployed internationally in the context of disaster management and response to medical emergencies. For example, during the COVID-19 pandemic, Cuba health workers were deployed to assist in treating COVID-19 patients in Italy ^12^. In addition during the Ebola outbreak in West Africa, Cuba was among the countries that sent healthcare workers who actively participated in patient care in Sierra Leone and Guinea ^13^.

The design and implementation of HRH interventions play a crucial role in their success. Analyzing the implementation experiences of these interventions is crucial for deriving valuable insights into their development and delivery mechanisms. While various studies have described Cuban health cooperation programs in general, there has been no review which has specifically focused on the design and implementation experiences of these increasing international Cuba health cooperation programs across various LMICs. In addition, one previous review primarily emphasized on the short-term missions of sending healthcare workers, with little attention to how these programs are conceived, designed, and implemented ^14^. This scoping review examines the design and implementation experiences of Cuban health cooperation programs across LMICs, focusing on the emergence, design, implementation experience, utility and outcome of these collaborative programs.

## Methods

We conducted a scoping review on the implementation experiences of Cuban health cooperation programs in LMICs. We developed a priori protocol and registered in the Open Science Framework ^15^. We conducted the review guided by the Joanna and Briggs Institute (JBI) guidelines for scoping reviews ^16^ and adhered to the Preferred Reporting Items for Systematic Reviews and Meta-Analyses extension for Scoping Reviews (PRISMA-ScR) checklist ^17^.

### Literature search

We searched for relevant articles across four databases: PubMed, Web of Science, Scopus, and Google Scholar. We developed a comprehensive search strategy in consultation with a research librarian. The key search terms were derived from the research question. An example of the search strategy on PubMed can be found in additional files ^17^. The last search was conducted on May 31, 2024. All identified articles were exported to Zotero software for managing references ^18^.

### Eligibility criteria

We included all articles describing Cuban health cooperation programs in LMICs including, primary research articles, commentaries, opinion papers, and editorials published between 2000 and 2024. We excluded articles not published in English, from high-income countries and study protocols, systematic reviews, book chapters, and commentaries with insufficient information. As outlined in the inclusion and exclusion criteria shown in Table 1. We selected eligible articles guided by the Population-Concept-Context (PCC) framework as outlined below ^18^:

**Table 1.** Showing the inclusion and exclusion criteria.

Criteria	Inclusion	Exclusion
Population	Cuban-health cooperation	Non-Cuban health cooperation
Context	LMICs	Non-LMICs
Language	English	Non-English
Access	Access to full- text article	No access to full-text article
Publication years	No limit

**Population**: We included articles reporting on Cuban health cooperation
**Concept**: We included articles that described the implementation of Cuban health cooperation programs
**Context:** We included articles from Low and Middle-income countries, including those describing Cuban health cooperation programs in single countries and multi-countries

The outcomes of interest for this review included the emergence of the Cuban health cooperation programs, their intended goals, the implementation processes, their utility, and the outcomes and lessons learned. 

### Study selection

We uploaded all references compiled in Zotero to Covidence ^19^, a web-based software used for systematic review and allows blinded screening by multiple reviewers. Two independent reviewers (FN and MN) selected articles by reviewing their titles, abstracts and full texts against the eligibility criteria. Discrepancies were resolved through discussions with the team and consultation with a senior reviewer (JN). Articles that met the inclusion criteria were included in the review.

### Data charting process and analysis

We extracted data using data from all included articles using a data extraction form in Microsoft Excel (2016) software. FN read all the articles to familiarize themselves with the data and extracted content based on the research objectives. We analyzed the data thematically and organized the data into categories, used basic coding and frequency counting, and synthesized the data to present review findings in narrative forms and tables

## Results

### Search results

The search across four databases: PubMed, Scopus, Web of Science and Google Scholar, yielded 3509 articles. After excluding 522 duplicates, we screened the remaining 2986 articles by title and abstracts and excluded 2804 articles at this stage. We subjected 175 articles to full-text screening and included 71 articles in the review. The PRISMA flow diagram showing the selection process is provided in extended data ^17^.

### Characteristics of included articles

We included 71 articles. Eighty per cent (80%) of these articles reported on the emergence of the Cuban health cooperation programs. All articles described the design and implementation process of these programs. Thirty-five per cent (35%) were from South America, twenty-one per cent (21%) were from Africa, thirteen per cent (13%) were from Pacific Islands and thirty-one per cent (31%) described the programs in multiple countries. Thirty-two per cent of the articles included were primary research articles, twenty per cent (20%) were commentaries, ten per cent (10%) were theses and 8% were reports. The articles were published between 2006 and 2023, most being published between 2016 and 2020. One-third of the articles employed qualitative study designs: including qualitative interviews, ethnography and explanatory case studies. Only four percent (4%) of the primary research studies used quantitative methods and three percent (3%) used mixed methods. Table 2 below shows a detailed description of the characteristics of included studies.

**Table 2.** Characteristics of studies on Cuban health cooperation programs.

Category	Details	Number(n) Percentage (%)
Publication Type	Primary research articles	23 (32%)
	Commentaries	14 (20%)
	Reviews	7 (10%)
	Thesis	6 (8%)
	Reports: project reports, evaluation reports	6 (8%)
Study Design	Qualitative studies	24 (34%)
	Quantitative studies	3 (4%)
	Mixed methods	2 (3%)
Year of Publication	2006–2010	21 (30%)
	2011–2015	20 (28%)
	2016–2020	24 (34%)
	2021–2023	7 (10%)
Regions	**South America** Brazil, Venezuela, Haiti, Bolivia, Chile, Guatemala, Ecuador	25 (35%)
	**Africa** West Africa: Nigeria, Sierra Leone, Ghana, Cape Verde Eastern Africa: Kenya, Uganda Southern Africa: South Africa	15 (21%)
	**Oceania** Timor Leste, Kiribati, Fiji, Solomon Island, Tuvalu	9 (13%)
	**Multi-country**	22 (31%)

## Emergence and goals of Cuban health cooperation across various LMICs

Most of the articles (80%) reported on the emergence and goals of Cuban health cooperation programs in the respective countries. The main reasons that contribute to the establishment of Cuban health cooperation programs in various countries included health workforce shortages and maldistribution, inequalities in health service provision in the public sectors and rural areas, inadequate local training capacity for medical students, government agenda prioritizing the attainment of universal health coverage (UHC), and provision of support during public health emergencies.

### Health workforce shortages

Health workforce shortages were the most reported reason for countries to enter into collaborative health programs with Cuba which would involve importing Cuban healthcare workers. Countries including South Africa, Timor Leste, Kiribati, Solomon Island, Brazil and Venezuela are reported to have lacked sufficient health workforce to provide services for their citizens ^9, 20– 22^. Health worker shortages in these countries were reported to be due to emigration to other countries, insufficient training, underfunding of the health sector, poor management and an inadequate skill mix. In post-apartheid South Africa, for example, the majority of physicians were reported to have emigrated from the country, creating a critical shortage of physicians. Poor working conditions, an overwhelming disease burden, declining educational standards and limited professional development were reported to have contributed to the physician emigration from South Africa. The South Africa- Cuba health cooperation program entered in 1996 was intended to address immediate shortages of physicians ^22^. In the Pacific Islands, severe health workforce shortages were reported to have been driven by insufficient training opportunities, migration and attrition of healthcare workers. This led to the country to enter into a bilateral health assistance programs with Cuba in 2002 to address the shortages ^21^.

### Health workforce maldistribution

Inequalities in healthcare workers distribution between rural and urban and between public and private sectors led various countries to enter into collaborative health programs with Cuba and import Cuban health workers. These disparities were reported to have resulted from poor resources and few incentives to attract healthcare workers in rural settings in these countries. Additionally, most graduating health workers in these countries were unwilling to work in rural areas ^23, 24^. A study conducted in Brazil reported that many medical graduates preferred to work in urban areas and with comparatively higher salaries, leaving rural settings without adequate medical doctor coverage. These resulted in the ‘ *Mais Medicos’* (More Doctors) Program between Brazil and Cuba which aimed at addressing these inequalities and providing health services in underserved urban and rural areas ^25^. In South Africa, the importation of Cuban doctors was done to provide doctors to be deployed in rural areas and public health sectors to provide services to underserved populations ^20^.

### Government agenda in prioritizing universal health coverage

In various countries, it was reported that government efforts to prioritise the provision of healthcare services for all citizens as part of efforts to attain UHC led to the importation of Cuban health workers. This was the case for Kenya, South Africa, Venezuela, Timor Leste and Solomon Island ^22, 26, 27^. A study in Venezuela recorded that in 1999, Venezuela rolled out a new constitution that redefined health as a fundamental right of citizens and a government's responsibility and mandate, establishing a universal, integrated, decentralized and participatory public healthcare system with full government financing. However, the country lacked sufficient healthcare workers to meet this objective. This led to the first health cooperation agreement with Cuba, importing Cuban physicians to work in underserved Venezuelan neighbourhoods, under the *Barrio Adentro* (Into the Neighbourhood) program ^26^. In Kenya, the importation of Cuban medical specialists was intended to address service delivery gaps at the county level and strengthen PHC services in line with the government development agenda in 2017 ^27^.

### Response to disasters and medical emergencies

Natural disasters and medical emergencies such as Ebola, the COVID-19 pandemic and earthquakes are case scenarios that have resulted in countries entering into collaborative health programs with Cuba, resulting in the importation and utilization of Cuban healthcare workers in various countries. During the Ebola outbreak in West Africa, Cuba was among the first countries to respond to the WHO's call for medical collaboration in response to the medical emergence and social disaster, to provide direct healthcare support during the pandemic ^28^. While most of these initiatives began as purely humanitarian aid, they often led to formalized bilateral agreements. This was the case in Haiti, where Cuban doctors were sent for disaster relief after Hurricane George in 1998, and a formal agreement followed soon after ^29^.

### Disease eradication programs

Countries with a high disease burden have partnered with Cuba to help fight against diseases. In Nigeria, for example, Cuban healthcare workers worked in malaria eradication programs, contributing to knowledge transfer and capacity building ensuring that the prevention and eradication programs are carried on once the Cuban experts leave the country ^30^. While Nigeria has a high malaria burden, Cuba successfully eradicated malaria in 1973. This health cooperation was formalized through an agreement signed between Cuba and Nigeria in 2000, which aimed to train local healthcare workers, improve the quality of primary healthcare, and enhance preventive measures against malaria.

## Design and implementation process of Cuban health cooperation programs in LMICs

All 71 articles highlighted the design and implementation process of Cuban health cooperation with the respective countries. The Cuban health cooperation programs have been designed differently in different contexts. In most countries, the programs are designed as exchange programs involving the importation of Cuban healthcare workers into the collaborating countries and sending local students for training in Cuba. These exchange programs were implemented in Brazil, Venezuela, South Africa, Kenya, Timor Leste, Guatemala, Bolivia, Haiti, Nigeria and Ecuador.

### Provision of health services in collaborating countries

Across collaborating LMICs, Cuban healthcare workers have been deployed and utilized to provide specialized healthcare services mostly in primary healthcare settings including South Africa, Kenya, Gambia, Cape Verde, Angola, Djibouti, Equatorial Guinea, Nigeria, Ghana, DRC, Liberia, Brazil, Venezuela, Bolivia, Haiti, Guatemala, Timor Leste, Pacific Island (Kiribati, Solomon Island, Fiji, Tuvalu, Vanuatu) and Chile.

***Contracting and Deployment.*** Most of the countries deployed Cuban healthcare workers to work in rural and primary healthcare settings ^9, 22, 27, 31, 32^. In Brazil, for example, Cuban healthcare workers were deployed to work in marginalized and poor rural and peri-urban municipalities ^25^. However, in Timor Leste and Solomon Island, the Cuban healthcare workers were deployed to both National and sub-regional hospitals based on the needs assessment by the local governments ^9^.

Most countries including Kenya, Brazil, South Africa, Cape Verde and Timor Leste deployed medical specialists including family medicine, physicians, paediatricians, and anaesthetists ^27, 31, 33^. In other countries such as Venezuela, they deployed both medical specialists and other healthcare workers such as nurses, epidemiologists and dentists ^26, 34^. The Cuban healthcare workers are deployed on a contract basis varying from three-year contracts to one-year contracts. In Brazil, the Cuban specialists were offered three-year contracts while in South Africa and Bolivia, the contracts were for a duration of two years ^22, 33, 35^. In Timor-Leste, the collaborating agreement covering the health cooperation was renewed each year and was expected to run until all the Cuban-trained doctors were deployed ^9, 36^.

***Induction and pre-assessments.*** The Cuban healthcare workers were often required to undergo an induction to orient them on the country's health system structures and clinical protocols ^22, 33^. In South Africa, medical professional boards interviewed the Cuban doctors before their deployed to assess their clinical skills, knowledge and language competencies ^22^. In Brazil, they deployed Cuban doctors with who had previously worked in at least one international mission and a minimum of 15 years’ experience ^25^.

### Collaborating country students training in Cuba

As part of the health cooperation, collaborating countries sent local medical students for training at the Latin American School of Medicine (ELAM), in Cuba. Within these arrangements, most of the collaborating LMCs sent undergraduate students for six years of medical training, which involves a one-year premedical course to learn Spanish and basic sciences. This is then followed by the medical curriculum that mainly focused on community-based health determinants, disease prevention strategies and knowledge building and coping with poor resources in low-resource settings ^20, 37– 39^.

The students were primarily selected from disadvantaged backgrounds to create an obligation for them to return and serve in rural areas/among underprivileged populations in the long term, thus offering a sustained improvement in the public healthcare provision ^20, 22, 32, 39– 41^. In contrast, countries like Kenya sent qualified medical doctors for postgraduate training in Family Medicine, with a focus on primary healthcare. The goal was to improve local capacity in primary health services and strengthen the local primary healthcare system upon their return ^27^.

***Integration into service delivery upon return.*** Returning students were often required to undertake additional exams and internships before being registered to work in their home countries. In South Africa for example, the students returned during their fifth year of studies to complete 1to 3 years of additional training at a local university. Additionally, the students were required to take the South Africa Medical Board exams ^20, 23, 42^.

## Utility and outcomes of Cuban health cooperation programs

### Improved access to health service delivery in underserved areas

Across collaborating LMICs, Cuban health collaborative programs have primarily focused on providing health services in rural areas and bringing care closer to the residents through community visits and working in remote regions. They have also helped to deploy medical doctors and medical specialists in areas where the numbers are usually too few or absent. In addition, collaborating country health workers trained in Cuba are deployed in rural areas in the public health sectors, resulting in improved access to health care in these areas ^9, 20, 22, 32, 40, 41^. In Timor Leste, each returning medical doctor from training in Cuba signs a contract with the government committing to work in the public sector for at least six years ^9^. A study conducted in South Africa showed that 80% of Cuban-trained medical doctors preferred to work in primary healthcare, mostly in rural areas ^40, 42^. This generally led to increased access to healthcare services in the rural and underserved areas, making healthcare delivery more equitable. In Venezuela, the *Barrio Adentro* program saw a massive increase in access to public health services where up to 73% of the population was covered. Within one year of the program (2004–2005), the program recorded 150 million patient visits, of which 40% were home visits ^26^.

### Increase in health workforce numbers

Across collaborating LMICs, importation of Cuban healthcare workers has increased the number of healthcare workers, addressing health worker shortages, particularly in rural areas ^22, 33, 38, 41, 43– 45^. The training component of these programs has led to a more long-term and sustainable increase in the number of healthcare workers in collaborating LMICs. In the Pacific Islands, the return of trained local doctors from Cuba doubled the number of available medical doctors ^21^. In Timor Leste, new medical graduates increased the number of medical doctors by more than threefold compared to before the implementation of the program), ensuring the country had a sufficient pool of medical doctors to meet immediate health workforce targets ^36, 39, 46^.

### Improved health outcome indicators

Various articles reported on the impact of the Cuba health collaborative programs on health outcomes across various collaborating, LMICs. In a study conducted in Kiribati, the work of Cuban medical doctors in conducting birth delivery and caesarean sections, reported that infant mortality rates reduced by 80% following the arrival of the doctors, dropping from 50 to 9.9 per 1000 live births ^21^. Studies in Haiti, reported improvements in healthcare indicators following the collaborative program with Cuba, indicating a reduction in infant mortality rates, child mortality and maternal mortality rates ^29, 41^. In West Africa, studies reported that the presence of Cuban medical doctors led to a reduction of hospital-based case fatality rates from 80–90% to 24% in 6 months ^12^.

### Support local training and capacity building

Cuban health brigades participated in tutoring at local universities, developing curricula for various courses and establishing new schools in various collaborating LMICs. In Venezuela, the Cuban medical doctors participated in developing curricula and providing faculty members for the new national training program for comprehensive community physicians within six Venezuelan universities, a program that aimed at training physicians for public service and adopted a community-based education model ^26, 47^. In Timor Leste, the imported Cuban doctors inaugurated the first faculty of medicine at the National University, marking the beginning of in-country training for medical doctors ^9, 39^. Similarly, the imported Cuban doctors have been part of the creation of medical schools in the Gambia, Ethiopia, Guinea Bissau, Yemen and Guyana ^48, 49^.

### Client satisfaction

Assessments of Cuban health cooperation programs in different collaborating LMICs reported that the clients attended by the Cuban medical doctors reported very good satisfaction with the services received. A study conducted in Brazil reported that 94.6% of the clients were satisfied with the services received ^44^. This was reported to be attributed to the fact that the Cuban healthcare workers lived among the people, listened to them, were more committed to on-site work and stayed there longer, increasing trust among the patients and local communities ^50^.

### Professionalism, skills transfer and motivation

Various articles reported that Cuban healthcare workers deployed in collaborating LMICs approached work professionally and maintained high discipline and integrity. Most had good relationships with the local staff and could transfer skills through Continuous Medical Education (CME) seminars and training during ward rounds ^22^. Several articles reported that although their skills and experiences were not always entirely appropriate for the local disease environment, the Cuban doctors were willing to learn and adapt quickly to the new environment in host countries. Additionally, their experience working in the Cuban health system, which suffers from adequate resources (financial and pharmaceutical) challenges, always renders them well-suited to work in resources strained rural areas in LMICs ^31^

## Challenges faced in the implementation of Cuba health collaborative programs

### Language barrier

Across various collaborating LMICs, the language barrier was highlighted as a challenge that made doctors-to-patient interactions and their effective integration and collaboration with local doctors difficult ^21, 25, 50^. In South Africa, one of the main concerns was the competency with English, and they often required a translator to consult with patients. However, these doctors often learned the dominant local languages bypassing the need for translation into and from English ^31, 49^.

### Opposition against the Cuban professionals in collaborating countries

The importation of Cuban healthcare workers has faced opposition, especially from local medical professionals ^21, 33, 50– 52^. The local doctors based the opposition on inadequate language skills, inadequate knowledge of local diseases, and poor clinical standards by the Cuban doctors. They also based the opposition on inadequate consultation by the government and the perception of competition for their jobs. In Brazil, the medical professional bodies filed injunctions against the government's move to import Cuban specialists, citing the move as being unconstitutional. Their rejection of the Cuban healthcare workers was based on inadequate language skills and insufficient medical knowledge ^25, 33, 50^. In South Africa, there was a belief that Cuban doctors did not possess the relevant skills or knowledge and importing them deprived local doctors the job opportunities ^31^. In Bolivia and Venezuela, this resulted in healthcare workers' strikes and protests led by local medical associations who perceived the program as competition for their jobs. The protesting healthcare workers argued that the government should instead address the core issues in the public health system including underfunding, poor management and corruption ^11, 53, 54^. However, another study in South Africa indicated a positive perception from the medical doctors, who indicated that the Cuban doctors were posted mainly to rural hospitals and provided health services that may otherwise be unavailable ^49^.

### Discontinuity of programs due to government transitions in the collaborating countries

Changes in governments in the collaborating countries often led to abrupt discontinuation of the programs. In Brazil, for example, it was reported that the newly elected federal government in 2018, terminated the agreement with Cuba, leading to Cuba healthcare workers to return to their country. This sudden departure led to a significant shortage of physicians across the country, leaving a large and vulnerable population—primarily in the rural and marginalized municipalities—without adequate access to medical services ^55^.

## Discussion

Our review findings showed that Cuban health cooperation programs have been implemented across many LMICs in South America, Africa, Southeast Asia and the Pacific. Health worker shortages particularly in the rural and underserved regions, governments' agenda to achieve universal health coverage, inadequate local training capacity and response to medical emergencies were among the main reasons different countries instituted health cooperation programs with Cuba ^9, 20– 25, 28– 30^. The main reported decision makers in signing these health cooperation programs agreements are the top government officials, especially the president and Ministry of Health officials ^11, 21, 23, 36, 45^. Most programs are designed as exchange programs, which involve the importation of Cuban healthcare workers to provide services and sending local students for training in Cuba. The implementation differed among different countries in terms of the duration, deployed cadres, distribution and allocation.

Health worker shortages are one of the main reasons for LMICs to get into health collaborative programs with Cuba. These health cooperation programs are aimed at filling in gaps in health service delivery, particularly in the public sector and rural areas ^21, 33, 43, 56^. In addition, as many LMICs strive to attain universal health coverage (UHC), the need for an adequate, skilled, and evenly distributed health workforce has emerged. Consequently, different governments have aligned their political agendas intending to achieve UHC, aiming to ensure equitable access to quality healthcare for their citizens. Countries such as Brazil, Timor-Leste, South Africa, and Kenya have established health cooperation programs within the context of UHC aiming to deploy additional qualified healthcare workers to underserved areas, thereby improving access to healthcare service delivery ^9, 20, 27^. The health collaborative programs allowing for importation of additional and often specialized health workers have assisted these countries in addressing their respective health agenda.

Medical emergencies and natural disasters, such as the COVID-19 pandemic, Ebola outbreaks, earthquakes and hurricanes, contribute to significant shocks in health systems globally. As demonstrated during the COVID-19 pandemic, many health systems were highly strained, placing an immense burden on frontline healthcare workers and stretching the capacities of most countries ^57^. The need for health system with an adequate health workforce proved paramount to meet the care demands. In response, many countries imported Cuban healthcare workers in these contexts. While the work of Cuban healthcare workers in such contexts is primarily in the form of medical missions and humanitarian response; bilateral collaborative agreements have been formalized following these missions ^29^. Cuban health cooperation programs have also emerged in various countries within the framework of specific disease eradication initiatives, such as malaria eradication programs in Nigeria ^30^.

The review findings indicate that the establishment of the bilateral agreements depended on the political of orientation of the collaborating government with key decisions made by high-ranking government officials: including the executive (presidents) and Ministry of Health officials ^11, 21, 23, 36, 45^. This high-level political interest has facilitated the successful implementation of these programs by ensuring authority and support. However, the data suggest that changes in government often lead to the abrupt discontinuation of these programs, limiting their anticipated benefits ^55^. Additionally, the findings reveal a lack of collaborative decision-making in health cooperation agreements, with key stakeholders—such as healthcare workers and medical professional bodies—frequently excluded from the process. This exclusion has led to opposition, including health worker strikes and legal challenges against these government initiatives in Brazil, South Africa, and Bolivia ^23, 52^.

More attention is needed at both the policy and implementation levels to support the integration of returning health professionals into local healthcare systems. In most countries, it was reported that returning students had to undertake additional training or internships. While this helped ensure equivalence with locally trained doctors, it failed to recognize the benefits of the community-oriented training developed in Cuba, delaying their clinical debut and potentially undermining their commitment to serve in rural and primary healthcare settings ^20^. It is also crucial to ensure that the training provided in Cuba aligns with the local context of the collaborating country. This involves evaluating whether the medical training aligns with the specific health needs of the collaborating country. Such proper alignment will prevent the need for additional training and ensure returning doctors have the necessary skills and knowledge to practice effectively ^31, 46, 56^.

Cuban health cooperation programs have contributed to an increase in the number of healthcare workers, particularly in rural and underserved areas. While these initiatives were primarily short-term, the training components facilitate a more sustainable, long-term expansion of the health workforce, thereby enhancing access to healthcare service delivery. However, the impact on health service delivery and outcomes was more significant in countries with more extensive programs, such as Brazil and Venezuela. Conversely, in countries like Kenya, where they deployed a few doctors and trained just a handful, the increase in human resources for health (HRH) was less sustained. Short-term programs may achieve more substantial outcomes if they focus on capacity building rather than direct service delivery, as was the case in Nigeria.

## Conclusion

The findings underscore the need for more collaborative decision-making in the planning and implementation of health cooperation programs. Particularly involving key stakeholders, such as healthcare workers and professional bodies, ensuring better-aligned program objectives and smoother implementation. Proper planning, including conducting needs assessments to inform the distribution and allocation of the imported health care workers was a key factor to maximize these programs. Political will and support are a key determinant in the establishment and implementation of these programs. Governments should establish policies and frameworks to ensure that these programs are integrated into broader health systems goals and align with national health priorities. Deployment of healthcare workers in rural and underserved areas increased equitable access to healthcare services and is likely to advance progress towards UHC. Targeted training programs for students in rural areas should be prioritized, fostering a well-trained workforce committed to serving the underserved areas. Future research should focus on longitudinal studies to evaluate the long-term impact of Cuban health programs on health systems in LMICs, especially looking into sustainability and long-term effectiveness.

## Ethics and consent

Ethical approval and consent were not required.

## Data Availability

This study used existing literature data. All data underlying the results are available as part of the article and no additional source data are required. Havard Dataverse: Replication Data for: Experiences with the Implementation of Cuban Health Cooperation Programs in Low and Middle-Income Countries: A Scoping Review https://doi.org/10.7910/DVN/TCJTE0 ^17^. This project includes the following extended data: 1.  Characteristics of Included articles.docx 2.  PRISMA-ScR Flow Diagram.docx 3.  PRISMA_ScR_Checklist _Additional file 3.docx 4. Sample search strategy.docx Havard Dataverse: PRISMA ScR Checklist for “Experiences with the Implementation of Cuban Health Cooperation Programs in Low and Middle-Income Countries: A Scoping Review” https://doi.org/10.7910/DVN/TCJTE0 ^17^. Data are available under the terms of the Creative Commons Attribution 4.0 International license (CC-BY 4.0).
