# Supplementary material for: Experiences and Outcomes of the Implementation of Cuban Health Cooperation Programs in Low and Middle-Income Countries: A Scoping Review
Source: Wellcome Open Res. 2025 Aug 29;10:167. Originally published 2025 Mar 28. [Version 2] doi: 10.12688/wellcomeopenres.23844.2 (PMC12107231; doi:10.12688/wellcomeopenres.23844.2)
Supplement: Peer review history

# Reviewer report: Reviewer response for version 2

**DOI:** 10.21956/wellcomeopenres.27317.r130952

**Published:** 2025-09-16

**Authors:** Stefan Swartling Peterson

**Affiliations:** 1 Makerere University, Kampala, Central Region, Uganda

I am satisfied with manuscript.

Are the rationale for, and objectives of, the Systematic Review clearly stated?

Yes

Is the statistical analysis and its interpretation appropriate?

Not applicable

If this is a Living Systematic Review, is the ‘living’ method appropriate and is the search schedule clearly defined and justified? (‘Living Systematic Review’ or a variation of this term should be included in the title.)

Not applicable

Are sufficient details of the methods and analysis provided to allow replication by others?

Yes

Are the conclusions drawn adequately supported by the results presented in the review?

Yes

Reviewer Expertise:

NA

I confirm that I have read this submission and believe that I have an appropriate level of expertise to confirm that it is of an acceptable scientific standard.

# Reviewer report: Reviewer response for version 2

**DOI:** 10.21956/wellcomeopenres.27317.r130954

**Published:** 2025-09-16

**Authors:** Roger A Atinga

**Affiliations:** 1 University of Ghana Business School, Accra, Ghana

The revision is satisfactory and I have no further comment

Are the rationale for, and objectives of, the Systematic Review clearly stated?

Partly

Is the statistical analysis and its interpretation appropriate?

Not applicable

If this is a Living Systematic Review, is the ‘living’ method appropriate and is the search schedule clearly defined and justified? (‘Living Systematic Review’ or a variation of this term should be included in the title.)

Yes

Are sufficient details of the methods and analysis provided to allow replication by others?

Yes

Are the conclusions drawn adequately supported by the results presented in the review?

Partly

Reviewer Expertise:

Health policy and management, health systems research

I confirm that I have read this submission and believe that I have an appropriate level of expertise to confirm that it is of an acceptable scientific standard.

# Reviewer report: Reviewer response for version 1

**DOI:** 10.21956/wellcomeopenres.26303.r121470

**Published:** 2025-05-26

**Authors:** Prudence Ditlopo

**Affiliations:** 1 Centre for Health Policy, School of Public Health, University of the Witwatersrand, Johannesburg, Gauteng, South Africa

This paper conducted a scoping review to provide a detailed mapping of the implementation of the Cuban Health Cooperation Programs as experienced in LMICs. Following a review of 71 peer-reviewed articles, the paper provides valuable insights into the emergence, nature and rationale for these programs across countries, the contribution of the Cuban doctors to host countries, challenges experienced by these programs and lessons learnt. The paper is well-written, and I only have minimal suggestions to improve it further for indexing:

1. In the Methodology section, was there a particular reason for excluding systematic reviews in the search strategy?
2. In the Discussion section:
  1. In terms of the findings on the emergence of these programs due to health workforce shortages and maldistribution, the authors could have reflected on the emerging struggles to absorb newly trained doctors or even employ them due budget constraints in some countries like South Africa.
  2. The authors could have reflected on some of the eminent limitations of a scoping review – such as limited critical appraisal of the quality and risk of bias of the studies included, leading to conclusions potentially being based on low-quality or methodologically weak studies. The authors could have also explicitly stated that one of the limitations of the review is that they only focused on English papers.
3. I have a few editorial suggestions as listed below:
  1. In page 7, first paragraph on the right, the authors wrote that “ *The Cuban healthcare workers are deployed on a contract basis varying from three-year contracts to one-year contracts.”* Slightly revise this as follows: *“The Cuban healthcare workers are deployed on a contract basis varying **from one-year contracts to three-year contracts**.”*
  2. In page 7, second paragraph on the right, the authors wrote that *“In Brazil, they deployed Cuban doctors **with** who had previously worked in at least one international mission and a minimum of 15 years.”* Cancel **‘with’**.

Are the rationale for, and objectives of, the Systematic Review clearly stated?

Yes

Is the statistical analysis and its interpretation appropriate?

Not applicable

If this is a Living Systematic Review, is the ‘living’ method appropriate and is the search schedule clearly defined and justified? (‘Living Systematic Review’ or a variation of this term should be included in the title.)

Not applicable

Are sufficient details of the methods and analysis provided to allow replication by others?

Yes

Are the conclusions drawn adequately supported by the results presented in the review?

Yes

Reviewer Expertise:

health systems and policy, specifically human resources for health research, health workforce practice, incentives and motivation, workforce policy analysis, health workforce management and governance.

I confirm that I have read this submission and believe that I have an appropriate level of expertise to confirm that it is of an acceptable scientific standard.

# Reviewer report: Reviewer response for version 1

**DOI:** 10.21956/wellcomeopenres.26303.r121475

**Published:** 2025-05-26

**Authors:** Roger A Atinga

**Affiliations:** 1 University of Ghana Business School, Accra, Ghana

There are no explicit objectives of the within which to locate the results. Authors have presented a laundry of results which are not clearly connected by underlying objectives.

 Given the coverage of the findings, the title should be changed to "Experiences and impact with the Implementation of Cuban Health Cooperation Programs in Low and Middle-Income Countries: A Scoping Review"

 Some of the thematic areas in the results needs strengthening. These include

 Health workforce malnutrition: rather than highlighting elements of maldistribution, authors should highlight how the Cuban program has helped mitigate this phenomenon.

 Government agenda in prioritizing universal health coverage: authors should focus analysis on how the Cuban is helping governments achieve UHC agenda.

 The recommendations should be adequately tied to issues identified in the findings

Are the rationale for, and objectives of, the Systematic Review clearly stated?

Partly

Is the statistical analysis and its interpretation appropriate?

Not applicable

If this is a Living Systematic Review, is the ‘living’ method appropriate and is the search schedule clearly defined and justified? (‘Living Systematic Review’ or a variation of this term should be included in the title.)

Yes

Are sufficient details of the methods and analysis provided to allow replication by others?

Yes

Are the conclusions drawn adequately supported by the results presented in the review?

Partly

Reviewer Expertise:

Health policy and management, health systems research

I confirm that I have read this submission and believe that I have an appropriate level of expertise to confirm that it is of an acceptable scientific standard, however I have significant reservations, as outlined above.

# Reviewer report: Reviewer response for version 1

**DOI:** 10.21956/wellcomeopenres.26303.r121474

**Published:** 2025-04-09

**Authors:** Stefan Swartling Peterson

**Affiliations:** 1 Makerere University, Kampala, Central Region, Uganda

Thank you for summarising this experience across the world.  

 I have a few questions which may contribute to further clarity:

 1)    This review covers LMICs and several continents. Are you able to comment on L vs M ICs, and sending these medical staff to countries where Spanish is spoken vs not? 

 2)    Relatedly do you think only summarising English language literature may introduce a bias? 

 3)    Similarly, are there differences in experience depending on the objective of the collaboration, e.g. gap filling, capacity development, etc?  Is this type of collaboration more suited to one objective than the other?

 4)    In the abstract you make reference to the objective from the Cuban side as “to boost their economy”.  I do not find this statement substantiated in the review,which does not seem to present a “Cuban perspective”.  Has the Cuban objective been the same over the entire period, or has it evolved?

 5)    Relatedly I miss a section on the financial aspects of these collaborations. In Uganda the Cuban doctors are rumoured to be paid USD 1500 per month plus fringes – more than Ugandan doctors. On the other hand hearsay says that the Cuban government keeps some/most of this money, only paying a fraction to the actual health worker.  Is there any literature on these aspects? From the Cuban Government and Cuban Health worker point of view, respectively?

 6)    In several places you make reference for this arrangement being a response to health worker shortages. Meanwhile literature is now starting to emerge of unemployed local health workers due to lack of absorptive/financing capacity. I would love to see a discussion of how to reconcile these perspectives in a way forward section of the discussion. 

 See e.g. Hutchinson E, Kiwanuka S, Muhindo R, Nimwesiga C, Balabanova D, McKee M, Kitutu FE. The paradoxical surplus of health workers in Africa: The need for research and policy engagement. Int J Health Plann Manage. 2024 May;39(3):956-962. doi: 10.1002/hpm.3745. Epub 2024 Jan 9. PMID: 38193753.

 7)    For the methods section a methodological reference would be nice.

 8)    In Table 2 only the Regions summarize to 100% - what are the missing Publication types, designs and study periods?

 Thanx again for this useful addition to the literature.

Are the rationale for, and objectives of, the Systematic Review clearly stated?

Yes

Is the statistical analysis and its interpretation appropriate?

Not applicable

If this is a Living Systematic Review, is the ‘living’ method appropriate and is the search schedule clearly defined and justified? (‘Living Systematic Review’ or a variation of this term should be included in the title.)

Not applicable

Are sufficient details of the methods and analysis provided to allow replication by others?

Yes

Are the conclusions drawn adequately supported by the results presented in the review?

Yes

Reviewer Expertise:

NA

I confirm that I have read this submission and believe that I have an appropriate level of expertise to confirm that it is of an acceptable scientific standard, however I have significant reservations, as outlined above.
